# Comparison of Intranasal Steroid Application Using Nasal Spray and Spray-Sol to Treat Allergic Rhinitis: A Preliminary Investigation

**DOI:** 10.3390/jcm12103492

**Published:** 2023-05-16

**Authors:** Antonio Moffa, Lucrezia Giorgi, Luca Carnuccio, Rodolfo Lugo, Peter Baptista, Manuele Casale

**Affiliations:** 1 Integrated Therapies in Otolaryngology, Fondazione Policlinico Universitario Campus Bio-Medico, 00128 Rome, Italy; 2 School of medicine, Università Campus Bio-Medico di Roma, 00128 Rome, Italy; 3 Unit of Measurements and Biomedical Instrumentation, Department of Engineering, Università Campus Bio-Medico di Roma, 00128 Rome, Italy; 4 Department of Otorhinolaryngology, Grupo Medico San Pedro, Monterrey 64660, Mexico; 5 Department of Otorhinolaryngology, Clinica Universidad de Navarra, 31008 Pamplona, Spain; 6 ENT Department, Al Zahra Private Hospital Dubai, Dubai 23614, United Arab Emirates

**Keywords:** nasal device, Spray-sol, nasal spray, beclomethasone dipropionate, allergic rhinitis

## Abstract

Allergic Rhinitis (AR) is a chronic inflammatory disease of sino-nasal mucosa, is IgE-mediated, and affects 10–40% of the global population. This study aimed to compare the efficacy of nasal administration of Beclomethasone Dipropionate (BDP) delivered via Spray-sol with nasal spray in patients suffering from AR. We included 28 AR patients assigned to one of the two following treatments: the Spray-sol group (BDP via Spray-sol) (*n* = 13) and the spray group (BDP using a common nasal spray) (*n* = 15). Both treatments were administered twice daily for 4 weeks. A nasal endoscopy evaluation and Total Nasal Symptom Score were performed at baseline and after treatment. The Spray-sol group showed better results than the spray group regarding nasal endoscopy (edema, *p* < 0.01; irritation, *p* < 0.01; secretion, *p* < 0.01) and nasal symptoms (nasal congestion, *p* < 0.05; rhinorrhea, *p* < 0.05; sneezing, *p* < 0.05; and total score, *p* < 0.05). No side effects were recorded. These data supported the fact that the use of BDP delivered with Spray-sol is more effective than BDP nasal spray in AR patients. Further studies are needed to confirm these encouraging results.

## 1. Introduction

Allergic Rhinitis (AR) is a chronic inflammatory disease of sino-nasal mucosa and is IgE-mediated. Usually, AR patients complain of rhinorrhea, nasal obstruction, sneezing, and itching [1]. It affects 10–40% of the global population [2], and its prevalence continues to increase worldwide. According to the “Allergic Rhinitis and its Impact on Asthma” (ARIA) guidelines, AR is now classified based on symptom duration (intermittent or persistent) and severity (mild, moderate, or severe). In particular, there are two main forms: “intermittent” AR, if symptoms are present less than 4 days per week or for less than 4 consecutive weeks, and “persistent” AR, when symptoms are present more than 4 days per week and for more than 4 consecutive weeks. Symptoms are classified as mild when patients can perform everyday activities (such as work or school) and have no sleep problems, while AR is categorized as moderate/severe if the symptoms significantly affect sleep or activities of daily living and/or if they are considered bothersome [3]. In addition to nasal symptoms, AR patients may also present with associated allergic conjunctivitis, non-productive cough, Eustachian tube dysfunction, and chronic sinusitis. The best treatment for AR is to avoid contact with inciting antigens. However, this is challenging to do, and it is necessary to use nasal and oral drugs. Although many pharmacological treatment options are available, two major drug types are commonly used today: antihistamines and corticosteroids. For patients with persistent moderate-severe (PM-S) AR, ARIA recommends as first-line treatment the daily use of Intranasal Corticosteroids (INCs) [3]. INCs have been shown to reduce nasal congestion, rhinorrhea, sneezing, and itching and can also relieve ocular symptoms.

Topical drugs reduce potential systemic side effects by acting directly on the nasal mucosa, so they are considered safer than orals. The most commonly observed side effects are burning, dryness, and epistaxis, generally overweighted by therapeutic benefits [4]. Among available INCs, Beclomethasone Dipropionate (BDP) is one of the most known and prescribed ones. Much evidence suggests that nasal nebulization is a more effective method of delivering topical medication than nasal spray because it provides a more excellent distribution of topical agents to the more profound and higher portions of the nasal cavities. Among the different nasal nebulization systems, there is a new nasal device called “Spray-sol” (BI4630R/REPA/rst patent and PCT/IB2014/065121), Brio Srl, Rome, Italy.

We started from the hypothesis that nebulization with Spray-sol can provide a more extensive and intense delivery of solutions to the nasal mucosa than sprays.

This study aimed to compare the efficacy of BDP administration via Spray-sol and nasal spray in AR patients regarding nasal endoscopic evaluation, nasal symptoms, and compliance with treatment.

## 2. Materials and Methods

This monocentric, open-label study was conducted at the Unit of Integrated Therapies in Otolaryngology of the Fondazione Policlinico Universitario Campus Bio-Medico after the approval of the Ethics Committee (protocol code 93/20 PAR ComEt CBM approved on 17 December 2020).

Subjects between 18 and 65 years of age with a history of persistent AR were consecutively recruited after an ENT evaluation at the Unit of Integrated Therapy in Otolaryngology from 1 November 2020 to 31 January 2022. Inclusion criteria were persistent AR based on documented allergic sensitization at Prick Test (positivity defined by a pomphus diameter 3 mm larger than control) to one or more common allergens, such as betulaceae (birch), corylaceae (hornbeam), fagaceae (oak and beech), oleaceae (ash and olive), cypress, plane and pine, urticaceae and gramineae, dust mite, dogs’ and cats’ hair, and dandruff, within 12 months prior to enrolment, with a history of chronic nasal obstruction. Exclusion criteria were patients with diseases or dysfunction of the immune system; chronic rhinosinusitis with and without nasal polyposis; marked deviation of the nasal septum; upper respiratory tract infections in the two weeks before recruitment; congenital malformations of the airways; cystic fibrosis; and treatment with systemic corticosteroids and/or antibiotics in the two weeks before recruitment.

At enrolment, the following baseline parameters were obtained: sex, age, allergy history, nasal endoscopic evaluation (Table 1), and a complete Total Nasal Symptom Score (TNSS) (Table 2). After enrolment, patients were assigned to one of the two following treatments:

- Spray-sol group: nasal administration of BDP (CLENIL 0.8 mg/2 mL suspension for nebulization, one single-dose vial 2 mL for aerosol) plus 3 mL of isotonic saline solution, dividing the content equally between the two nostrils, twice a day (in the morning after breakfast and in the evening before bedtime) for four weeks. Patients used BDP with Spray-sol according to the following instructions:
  ○ remove one single-dose vial and heat it between your hands before administering the solution;
  ○ open the vial by turning the cap, connect it to the Luer-Lock syringe, and aspirate the solution to be sprayed;
  ○ fix the Luer-Lock syringe by screwing it into the appropriate Spray-sol nozzle;
  ○ divide the solution contained in the syringe equally between the two nostrils;
  ○ before storing the device, rinse the syringe under running water and wash Spray-sol.
- Spray group: nasal administration of BDP solution using a common nasal spray (Rinoclenil 100 microgram nasal spray, suspension), one spray per nostril twice daily (in the morning after breakfast and in the evening before going to bed) for four weeks.
- The two groups recorded no statistically significant differences regarding age, BMI, nasal endoscopy, and TNSS.

Patients were assigned to the groups through consecutive enrolment, allocating odd-numbered patients to the Spray-sol group and even-numbered patients to the spray group, following the order of inclusion.

At the end of the 4-week treatment period, all the patients underwent a follow-up ENT examination with a nasal endoscopy evaluation, TNSS, and evaluation of the Visual Analog Scale (VAS) for treatment compliance.

### 2.1. Spray-Sol Characteristics

It is a nasal nebulizer characterized by the ability to nebulize high-viscosity substances similar to an aerosol with very low administration times (10 s to 5 cc of substance). It is portable and does not need electricity as it is attached to a Luer-Lok syringe.

This device has been designed to deliver aqueous solutions and drugs into nasal cavities. There are several studies in the literature on the use of Spray-sol with saline solution [5,6,7]; however, despite being used in clinical practice to deliver drugs, there are no studies on this topic.

### 2.2. Statistical Analysis

The results were collected in a Microsoft Excel spreadsheet and analyzed in R statistical software version 4.1.2 (R Foundation for Statistical Computing, Vienna, Austria). The results are expressed as mean and SD. The normal distribution was confirmed using the D’Agostino–Pearson normality test, and the Independent Sample *t*-Test with a two-tailed distribution was used to compare them. Our criterion for statistical significance was set at *p*-values of less than 0.05.

## 3. Results

At the end of our selection process, 30 patients were enrolled, but 2 patients were lost. As a result, 28 patients completed the follow-up (mean age 44.66 years old; range 24–73), 13 in the Spray-sol group and 15 in the spray group. At baseline, no statistically significant difference was found between the two groups for the nasal endoscopy findings (edema, irritation, secretion, and crustiness, *p* > 0.05) and the TNSS (nasal congestion, rhinorrhea, sneezing, nasal itching, *p* > 0.05). The demographic and baseline characteristics of study participants are shown in Table 3.

**Figure 1:**
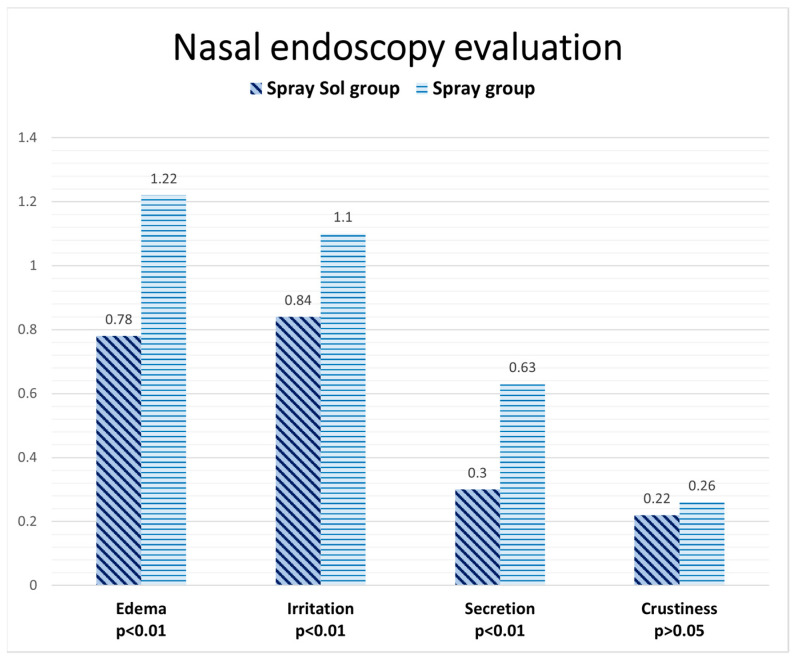
Nasal endoscopic evaluation data after four weeks of treatment: Spray-sol vs. nasal spray.

At the end of 4 weeks of treatment, regarding nasal endoscopy findings, the Spray-sol group showed better results than the spray group, reaching a statistically significant difference for the following investigated parameters: edema (0.78 ± 0.44 vs. 1.22 ± 0.35, *p* < 0.01), irritation (0.84 ± 0.55 vs. 1.10 ± 0.72, *p* < 0.01) and secretion (0.3 ± 0.48 vs. 0.63 ± 0.66, *p* < 0.01). Crustiness results were better in the Spray-sol group than the spray group without reaching statistical significance (0.22 ± 0.44 vs. 0.26 ± 0.49, *p* > 0.05). The results regarding the nasal endoscopic findings are shown in Figure 1

**Figure 2:**
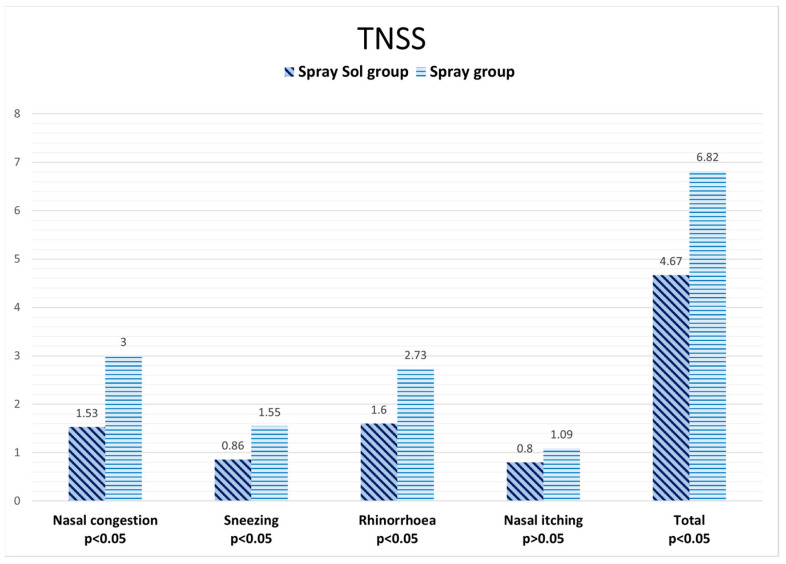
TNSS data after four weeks of treatment: Spray-sol vs. nasal spray.

At the end of 4 weeks of treatment, the Spray-sol group showed better results for TNSS than the spray group, reaching a statistically significant difference for the following investigated parameters: nasal congestion (1.53 ± 0.52 vs. 3.00 ±1.36, *p* < 0.05), rhinorrhea (1.60 ± 0.51 vs. 2.73 ± 1.27, *p* < 0.05), sneezing (0.86 ± 0.36 vs. 1.55 ± 0.52, *p* < 0.05) and the total score (4.67 ± 1.40 vs. 6.82 ±3.43, *p* < 0.05). Nasal itching results were better in the Spray-sol group than the spray group without reaching statistical significance (0.80 ± 0.41 vs. 1.09 ± 0.30, *p* > 0.05). The results regarding TNSS are shown in Figure 2.

No side effects related to the investigated treatments were recorded in either group during the study.

Regarding compliance, patients using BDP nebulized with Spray-sol reported higher results than those treated with nasal spray without reaching statistical significance (Spray-sol group: 6.25 ± 1.10; Spray group: 5.70 ± 1.10; *p* > 0.05).

## 4. Discussion

In clinical practice, for upper airways, the use of topical medication is usually recommended, as it allows for immediate treatment of the nose and neighboring structures with low medication dosages. Indeed, intranasal delivery can be used to both clean airways (“nasal hygiene”) by removing thick secretions (using isotonic or hypertonic saline solutions) and to administer drugs (hyaluronic acid, corticosteroids, antihistamines, antibiotics, etc.) to treat diseases [8].

There are many factors influencing the success of topical nasal treatment, such as delivery methods, gravity, obstructing anatomical structures, head positions, and the viscosity of the administrated substance. Additionally, the size of the nebulized particle plays a fundamental role in airway deposition: particles with a diameter of 1–5 μm are suitable for the lower airway; particles ranging from 5 to 10 μm are mainly deposited in the trachea and bronchi; while particles with a diameter > 10 μm are mostly deposited in the nose [5,9].

Among the different topical formulations, the most used ones are isotonic or hypertonic solutions, hyaluronic acid, steroids, or decongestants. To deliver these solutions, a wide variety of nasal devices are available, for example, syringes, nasal sprays, and nebulizers, each with their specific properties according to the particle size and administration technique. Investigations to determine the distribution pattern of different nasal devices are difficult and have been limited mainly through labor-intensive methodologies. The main difference between the available delivery devices is their ability to distribute the substance within the nasal cavities and the first airways. This aspect is crucial for choosing the most appropriate device. Although the topic is particularly practical and interests a wide range of physicians, the available literature is minimal [10]. According to the recent literature, nasal nebulizers showed better results over traditional nasal sprays in the distribution of fluorescein-impregnated saline solution, especially for the more profound and higher portions of nasal cavities [11,12,13].

Intranasal medications or other substances are usually administered by nasal drops, syringes, standard nasal sprays, and nebulizers [14].

A recent comparative study showed that Spray-sol produced particles with an average diameter (D) of 16 μm, similar to an aero-assisted nebulizer (Rinowash type, D = 16.5 μm), but significantly smaller than those produced by nasal sprays with a pressurized canister (D = 59.3 μm) and nasal sprays with a pump bottle (D = 34.7 μm) [15].

The particles produced by a nasal spray are big (typically between 50 and 100 µm in diameter [9]) and slow, so they usually settle in the anterior part of the nose. In contrast, those produced by nebulizers such as Spray-sol are smaller and faster, so they can also reach the posterior and upper regions of nasal cavities.

Thanks to the small diameter of the particles and the possibility to modulate the amount of substance, Spray-sol can not only quickly reach the inferior turbinate and the floor of the nasal cavities, but also the middle meatus and nasopharynx. In this way, Spray-sol guarantees a broader distribution of the BDP at the level of nasal mucosa than nasal spray, achieving better results. Consequently, the amount of substance in nasal cavities is increased, reaching the most posterior and upper regions of the nasal cavities. Moreover, Spray-sol does not require energy, has a low administration time (about 3–5 s for a complete and fine nebulization), is very easy to use, single-patient, and reusable. It is important to underline that the dispensing force of Spray-sol can be modulated according to the force applied to the syringe; therefore, in children, the rapidity of administration can be reduced when compared to adults.

In addition, Spray-sol can also be used to simultaneously deliver multiple types of saline solutions and drugs, corticosteroids, decongestants, and anesthetics. No adverse events related to the use of Spray-sol were described.

The theoretical advantage of Spray-sol is its ability to deliver a large volume of a drug (1 mL of 250 µg/mL) instead of the 32 µg delivered during one nasal spray.

Spray-sol can deliver a more significant amount of solution (5 mL for each application) than nasal spray (usually administered amounts of between 70 and 150 μL per puff [16]) thanks to the possibility of connecting a 10 mL syringe but also thanks to a more significantly capacious syringe via the Luer-Lock connection. These properties may help to increase patient compliance and improve clinical efficacy without increasing systemic side effects. Furthermore, Spray-sol is relatively inexpensive (€10 per device) and easily portable. Because it is disposable, patients can replace it when it becomes dirty or worn out, thus potentially limiting ongoing contamination during bacterial insults.

Despite the better deposition pattern of the nebulizers, the nasal spray is the dominant delivery device in the nasal drug delivery market, being inexpensive and simple to use. It represents the first-line treatment in many nasal conditions, typically reaching the nasal valve area, the region of maximum resistance to airflow [17].

To date, although Spray-sol has been designed to deliver not only saline solutions but also drugs, this is the first investigation reporting the effectiveness of Spray-sol in delivering a drug (corticosteroid). Subjects in this study received a short-term (4 weeks) treatment with topical BDP delivered via Spray-sol or nasal spray twice a day. At the end of therapy, the Spray-sol group showed a significant improvement in the TNSS (total score, nasal congestion, sneezing, and rhinorrhea) and endoscopic findings (edema, irritation, and secretion). Moreover, patients using BDP nebulized with Spray-sol showed better compliance than those treated with nasal spray. These results could be due to the greater distribution of BDP obtained by Spray-sol compared to nasal spray. Moreover, regarding compliance, patients preferred Spray-sol over nasal spray.

INCs are safe and effective both in the short and long term for AR patients. This study also corroborates previously published papers on the safety of INSI [18]. The literature on the side effects of Spray-sol is limited. However, no studies showed side effects [5,6,7,15,19,20].

Even if it has been demonstrated that nebulizers have a better deposition pattern, nasal spray still remains the dominant device in the nasal drug delivery market. Indeed, since it is low-cost and simple to use, it represents the first-line treatment in many nasal conditions, which typically involve the nasal valve area, the region of maximum resistance to airflow [17].

The small number of patients and the limited follow-up period represent the limits of this study. Further studies on a larger sample size are needed to confirm these encouraging results and to assess whether they decrease the need for additional surgery and oral steroid courses.

## 5. Conclusions

INCs are the mainstay of therapy for AR. Although four weeks is a short period in a disease as chronic as AR, the current study’s data support the fact that the use of BDP delivered with Spray-sol is more effective than BDP nasal spray in AR patients. Further studies are needed to confirm these encouraging results.

## Figures and Tables

**Table 1:** Nasal endoscopy-evaluation.

Nasal Endoscopy Evaluation	None	Mild	Moderate	Severe
Edema	0	1	2	3
Irritation	0	1	2	3
Secretion	0	1	2	3
Crustiness	0	1	2	3

**Table 2:** The Total Nasal Symptom Score (TNSS).

TNSS	Likert SCALE: 0—No Symptoms; 1—Mild Symptoms; 2—Moderate Symptoms; 3—Severe Symptoms			
Nasal Congestion	0	1	2	3
Sneezing	0	1	2	3
Rhinorrhea	0	1	2	3
Nasal Itching	0	1	2	3

**Table 3:** Demographic and baseline characteristics of study participants.

	Spray-Sol Group (12 Patients)	Spray Group (14 Patients)
Mean age (yr)	45.52	44.80
Weight (kg)	82.30	83.00
Height (cm)	176.00	175.70
**Nasal endoscopy evaluation**		
Edema	2.2 ± 0.41	2.1 ± 0.32
Irritation	2.2 ± 0.41	2.1 ± 0.32
Secretion	1.33 ± 1.05	0.6 ± 0.52
Crustiness	0.26 ± 0.46	0.5 ± 0.53
**TNSS**		
Nasal congestion	1.57 ± 0.98	2.25 ± 1.16
Sneezing	1 ± 1	1.125 ± 0.83
Rhinorrhea	1.14 ± 1.06	1.125± 0.64
Nasal itching	0.71 ± 0.76	0.75 ± 0.71
Total score	2.58 ± 2.94	3.82 ± 3.52

## Data Availability

The datasets generated and/or analyzed during the current study are not publicly available due to confidentiality reasons but are available from the corresponding author upon reasonable request.
